# TABLET TOSCANA to Develop Innovative Organizational Models for Tele-Rehabilitation in Subjects with Congenital and Acquired Developmental Disabilities: A Wait-List Control Group Trial Protocol

**DOI:** 10.3390/jcm13144159

**Published:** 2024-07-16

**Authors:** Veronica Barzacchi, Gloria Mangani, Benedetta Del Lucchese, Valentina Menici, Clara Bombonato, Elena Beani, Enrico Biagioni, Ilaria Palla, Federico Posteraro, Leopoldo Trieste, Giuseppe Turchetti, Giuseppina Sgandurra, Giovanni Cioni, on behalf of Tablet Toscana Consortium

**Affiliations:** 1 Developmental Neurology and Neurorehabilitation Unit, IRCCS Stella Maris Foundation, 56126 Pisa, Italy; veronica.barzacchi@fsm.unipi.it (V.B.); gloria.mangani@fsm.unipi.it (G.M.); benedetta.dellucchese@fsm.unipi.it (B.D.L.); valentina.menici@fsm.unipi.it (V.M.); clara.bombonato@fsm.unipi.it (C.B.); elena.beani@fsm.unipi.it (E.B.); giovanni.cioni@fsm.unipi.it (G.C.); 2 Tuscan Ph.D. Programme of Neuroscience, University of Florence, 50121 Florence, Italy; 3 Ph.D. Programme in Clinical and Translational Sciences, University of Pisa, Via Roma 67, 56126 Pisa, Italy; 4 Department of Clinical and Experimental Medicine, University of Pisa, 56126 Pisa, Italy; 5 UOC Neuropsichiatria Infantile, ASL Toscana Nord-Ovest, 56126 Pisa, Italy; enrico.biagioni@uslnordovest.toscana.it; 6 Institute of Management, Scuola Superiore Sant’Anna, 56126 Pisa, Italy; ilaria.palla@santannapisa.it (I.P.); leopoldo.trieste@santannapisa.it (L.T.); giuseppe.turchetti@santannapisa.it (G.T.); 7 Rehabilitation Department, Versilia Hospital, AUSL Toscana Nord Ovest, 55049 Lucca, Italy; federico.posteraro@uslnordovest.toscana.it

**Keywords:** tele-rehabilitation, neurodevelopmental disorders, pediatric neurological rehabilitation, Information and Communication Technologies, organizational models, feasibility, COVID-19

## Abstract

**Background/Objectives:** In recent years, the advent of new technologies has fostered their application in neuro-psychomotor and language rehabilitation, particularly since the COVID-19 pandemic. Tele-rehabilitation has emerged as an innovative and timely solution, enabling personalized interventions monitored by clinicians. TABLET TOSCANA project aims to develop innovative tele-rehabilitation organizational models in children, adolescents and young adults with congenital and acquired developmental disabilities, using the Virtual Reality Rehabilitation System (VRRS) Home Kit and the MedicoAmico APP. **Methods:** The trial is designed according to the CONSORT statement guidelines. The project encompasses three phases: adapting the technologies for pediatric use, validating them through a wait-list study, and analyzing feasibility and effectiveness data to define new organizational models. A randomized wait-list-control study with 100 subjects aged 6 to 30 years will compare tele-rehabilitation versus prosecution of standard care. **Discussion:** Although literature highlights tele-rehabilitation benefits such as improved access, cost savings, and enhanced treatment adherence, practical implementation remains limited (i.e., the definition of standardized procedures). TABLET TOSCANA project seeks to address these gaps by focusing on multi-domain treatments for neurodevelopmental disabilities and emphasizing the integration of tele-rehabilitation into local health services. **Conclusion:** The project aims to improve the continuity and intensity of care through innovative models that integrate tele-rehabilitation into local health services. The results could inform healthcare policies and promote the development of innovative and collaborative models of care, paving the way for more effective and widespread tele-rehabilitation solutions and fostering collaborative networks among professionals.

## 1. Introduction

In the last few years, there has been a significant increase in the development and application of new technologies for functional rehabilitation in developmental disabilities [1,2]. This trend has been further accelerated by the COVID-19 pandemic [3], which led to the complete closure of both public and/or private rehabilitation services, known as lockdown, across Italy, including the region of Tuscany. Specifically, within Tuscany, a significant decrease was observed in non-urgent medical procedures for both diagnosis and treatment. This reduction encompassed rehabilitative interventions, such as physiotherapy, speech therapy, and neuropsychological therapy [4]. Among the various consequences of the COVID-19 pandemic, a significant negative indirect impact on the quality of life of children with neurodevelopmental disabilities and their families was observed, including COVID-19-related rehabilitation services closure [5]. A longitudinal Italian study conducted during this period investigated lockdown-related emotional and behavioral changes in the pediatric neuropsychiatric population, revealing the increase in psychiatric symptoms [6].

To deal with this emergency, following the recommendations of scientific societies within the field, including Italian Society of Child and Adolescent Neuropsychiatry (SINPIA), Italian Society of Physical and Rehabilitation Medicine (SIMFER) and Italian Society of Neurological Rehabilitation (SIRN), some healthcare facilities responded by initiating tele-rehabilitation treatments, even if on a limited scale [7], and telehealth in their clinical practice with encouraging results [8,9,10].

Tele-rehabilitation is designed by the American Telemedicine Association as the provision of rehabilitation services through Information and Communication Technologies (ICT) [11] and is conceived as a timely, intensive, and personalized intervention aimed to provide access to rehabilitation services monitoring by clinicians.

This innovative approach facilitates activities in an ecological and home-based environment, ensuring continuity of care both spatially, at a distance from the clinical center, and temporally, extending the care beyond the hospitalization period [12]. In addition, it offers substantial cost and healthcare time savings [13] for both families and the healthcare system, increasing frequency and adherence to therapy [14], as well as treatment dosage and intensity [15], while also maintaining high levels of patient’s satisfaction [14,16,17]. However, several barriers have been identified in studies, such as technical literacy, availability of technology, connectivity issues and access to a sufficiently fast internet connection [18,19]. Technical literacy, in particular, is often associated with age or socioeconomic status [20].

New technologies to be integrated into rehabilitation pathways are even more crucial in the pediatric population, considering the prolonged treatments of neurodevelopmental disabilities as life-long conditions and, consequently, the extended hospitalization periods. Tele-rehabilitation allows us to address these issues, providing continuous home-based rehabilitation, supporting in the meanwhile motivation, and leveraging high-intensity interventions as key training elements highlighted in the literature [3].

Tele-rehabilitation, indeed, according to recent feasibility studies [21,22,23], proved to be particularly suitable to support the treatment of children with neurodevelopmental disorders and their families in order to enable continuity of care by extending rehabilitation programs into home settings, fostering family involvement with the continuous monitoring of the clinical team. This was even more relevant during the critical period of the COVID-19 emergency, as the use of ICT proved indispensable for the uninterrupted care of patients and their families and, consequently, mitigate the adverse consequences of care disruptions on the short-, medium-, and long-term neuropsychological outcomes, stress levels, and overall quality of life for the entire family [24].

A recent systematic review [25] investigating key aspects of tele-rehabilitation protocols for children with neurodevelopmental or neurological disorders revealed a heterogeneous and evolving landscape regarding the technologies used, intervention protocols, treatment efficacy and parent role during tele-rehabilitation. Results underscored the necessity for further improvements, especially in developing comprehensive and multi-domain rehabilitation protocols, and encouraged a more active role of the caregiver during treatment. This emphasizes the importance of home-based and family-centered care, potentially leading to better clinical outcomes for children with neurological or neurodevelopmental disorders.

Among the ICT, virtual reality can provide realistic experiences offering enhanced feedback (auditory, visual and tactile) in a user-friendly enriched environment [26], promoting enhanced learning and recovery for patients [27]. Moreover, virtual reality technologies align with rehabilitation principles, emphasizing the importance of meaningful, specific, and repetitive tasks and gradually increasing task difficulty over time [28,29]. Studies have reported the effectiveness of virtual reality in adult rehabilitation [17,30,31] and, although in fewer numbers, in children with neurodevelopmental disorders [32,33], including in tele-rehabilitation settings [34,35,36]. In this context, among different virtual reality tools the Virtual Reality Rehabilitation System (VRRS) developed by Khymeia (Padua, Italy) represents a promising device for rehabilitation. This multi-domain and integrated ecosystem supports neurological, orthopedic, cognitive speech, cardiorespiratory, and postural rehabilitation settings, mainly in the adult population.

Within this framework, TABLET TOSCANA aims to develop innovative organizational models that foster the continuity of care through tele-rehabilitation for neuro-psychomotor and language functions using Khymeia technological devices in subjects with congenital and acquired developmental disabilities. Particularly, organizational dimensions, acceptability, the usability of proposed solutions, and the performance of the involved ICT, unrelated to the effectiveness of tele-rehabilitation, will be assessed as the primary objective of the study, differently from traditional literature. In addition, the secondary objective will be to evaluate the clinical effectiveness of the tele-rehabilitation intervention. We hypothesize that the TABLET TOSCANA project could establish a new organizational model to be implemented at the territorial level and integrated into routine clinical practice, improving the taking charge of the rehabilitation services for children with neurodevelopmental disabilities.

## 2. Materials and Methods

### 2.1. Study Design

**Figure 1:**
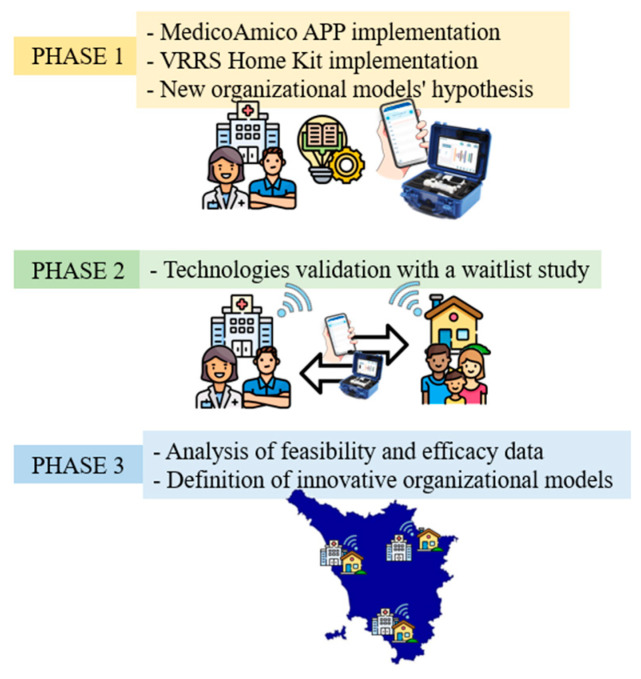
Three main phases of TABLE TOSCANA study. A synthetic overview of the three project phases detailed in the text: implementation of technological devices, hypothesizing in the meanwhile new organizational models integrating tele-rehabilitation within conventional care pathways; systems validation through a two parallel arm, wait-list study, recruiting participants with congenital and acquired neurodevelopmental disabilities; data analysis, both considering organizational dimensions, feasibility, and efficacy results.

The TABLET TOSCANA study will be carried out in 3 main phases (Figure 1) in order to implement new technological solutions for taking care and tele-rehabilitation, such as MedicoAmico PRO and Home Kit VRRS (Khymeia, Padua, Italy). The involved clinical centers include the Department of Developmental Neuroscience IRCCS Fondazione Stella Maris and the Azienda USL Nord-Ovest, dealing with subjects with congenital and acquired disorders.

#### 2.1.1. Phase I

**Figure 2:**
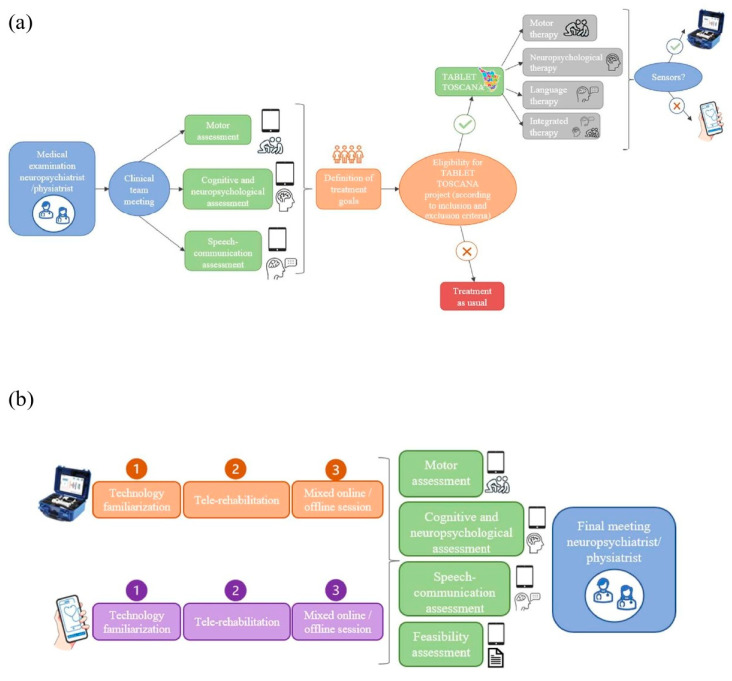
New organizational models’ hypothesis. (**a**) depicts the decision-making procedure with different steps for assigning patients to one of the study arms, according to the interdisciplinary clinical assessment (using standardized tests and technological devices) and the identified treatment goals. If the designed objectives could be achievable through technology and patients meet the inclusion criteria for the Tablet Toscana project, tele-rehabilitation pathways will be initiated. (**b**) illustrates the tele-rehabilitation pathway, involving an initial familiarization phase for patients and families with the system, followed by a 3-month treatment with mixed online and offline sessions. Thus, post-treatment evaluations will be conducted to gather efficacy and feasibility data.

The initial phase will involve the adaptation of both the MedicoAmico APP and VRRS Home Kit to the pediatric population’s specific needs since such tele-rehabilitation devices were originally designed for adults. The multidisciplinary clinical team, in collaboration with Khymeia bioengineers, in relation to their expertise, will meticulously analyze and implement the library of motor, cognitive and language exercises currently available in both systems to enhance their usability for the developmental age. Detailed descriptions of these technologies will be provided in the “Intervention” section below. In addition, during this phase, new organizational models for motor, cognitive and/or language tele-rehabilitation pathways of children and adolescents with congenital or acquired developmental disorders will be hypothesized, as detailed in Figure 2.

#### 2.1.2. Phase II

**Figure 3:**
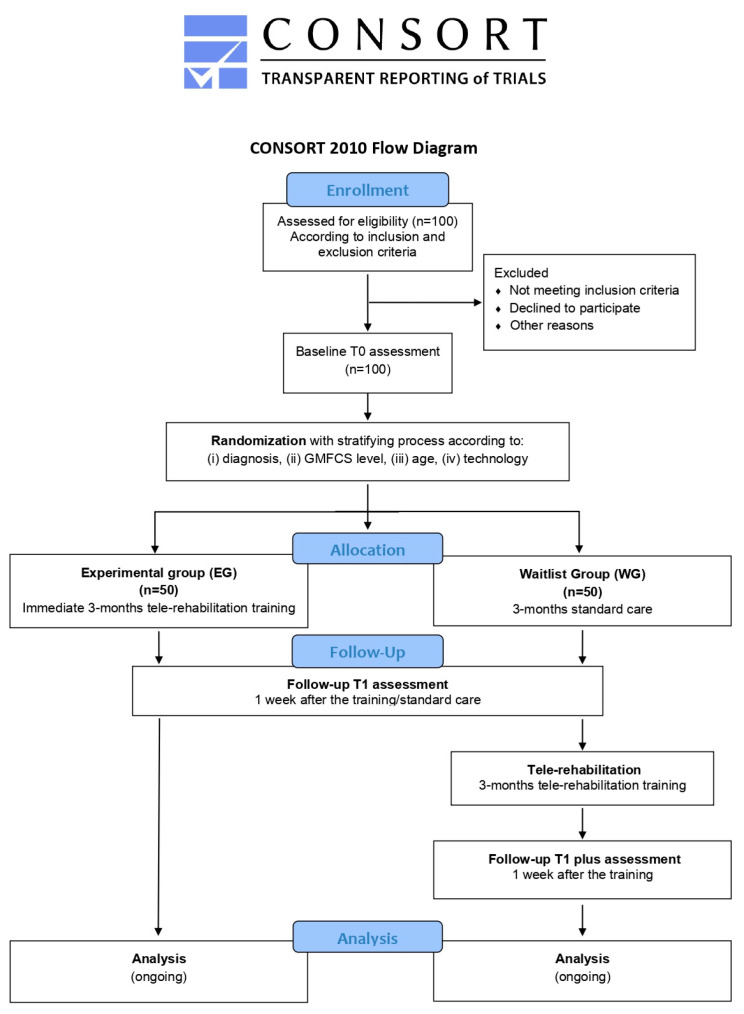
Flowchart of TABLET-TOSCANA study according to CONSORT guidelines. Graphical representation of the two parallel arms wait-list study, illustrating the pathway from the identification of the eligible patients, according to the inclusion and exclusion criteria, proceeding through a baseline assessment of cognitive, motor and speech-communication functions. Thus, a stratification process will be foreseen, considering different demographic, clinical and technological parameters, in order to foster the random assignment to one of the two experimental groups or to the control one. After a 3-month tele-rehabilitation program, with mixed online and offline sessions, post-treatment evaluations will be conducted.

During this phase, the validation of the implemented technologies will be conducted through a wait-list study (Figure 3) with two parallel arms. One hundred subjects (comprising 80 children and 20 young adults) will be recruited from the aforementioned clinical centers. The wait-list design study will ensure that all enrolled patients will be eligible for the experimental treatment, thus avoiding issues related to equipoise.

**Figure 4:**
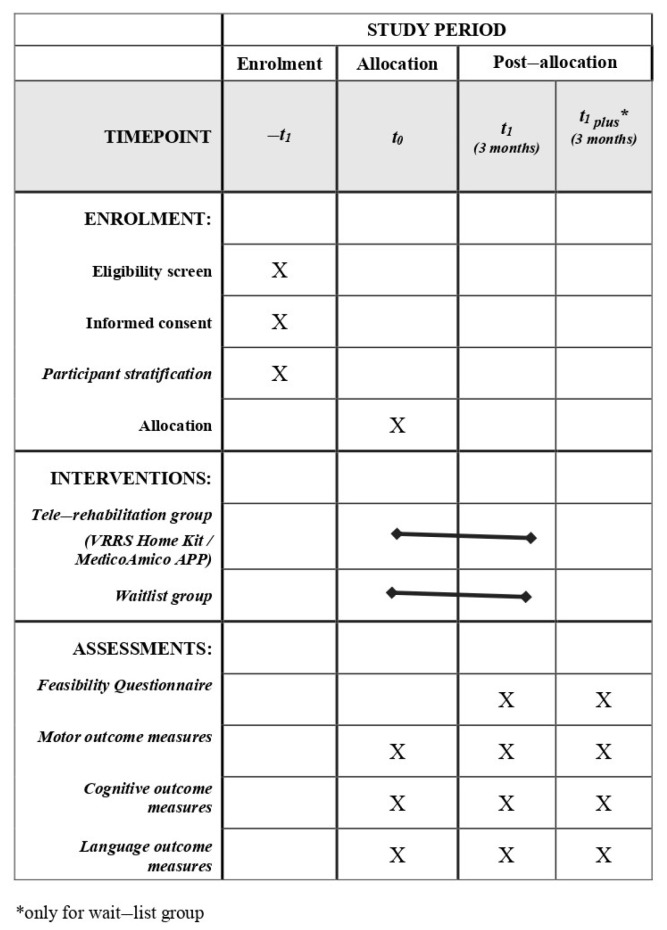
Spirit Figure. Schedule of study enrolment, interventions and assessments.

After the identification of eligible subjects according to inclusion and exclusion criteria (described below), participants will be stratified according to several criteria, including (i) diagnosis (congenital or acquired developmental disorder group), (ii) gross motor function classification system (GMFCS) level (GMFCS I–II/GMFCS III–IV), (iii) age group (6–10/11–15/16–20/21–30), (iv) and technology utilized (APP vs. Home Kit). Then, participants will be randomly assigned to the experimental group (EG)—receiving a 3-month tele-rehabilitation immediately—or the wait-list group (WG)—continuing traditional rehabilitation care for the same duration. All subjects will be evaluated before (T0) and after (T1) the 3-month intervention period (tele-rehabilitation or traditional rehabilitation), using standardized clinical tests. Moreover, participants enrolled in the WG will be re-evaluated at the end of the 3 months of tele-rehabilitation training (T1 plus). Finally, at the end of tele-rehabilitation training, ad-hoc questionnaires will be administered to the main stakeholders to assess the systems’ usability and acceptability. The program of enrolment, interventions and assessments designed according to SPIRIT guidelines are shown in Figure 4.

#### 2.1.3. Phase III

The third and final phase will involve the analysis of usability and acceptability data, considering the feasibility measures acquired. Moreover, efficacy data acquired from pre–post-intervention cognitive, motor, and language assessments will be considered. Furthermore, the research group will perform interviews with patients and parents to collect information regarding unmet needs in relation to the patient’s pathway. The healthcare professionals will be involved in a Focus Group to analyze the strengths and weaknesses of organizational models with and without technology. The interviews with patients and caregivers will be one-on-one and face-to-face. The interviews will be performed online. The interview will follow a semi-structured questionnaire that the research group will develop for the project. The Focus Group is a qualitative methodology based on discussion among participants managed by a moderator. The Focus Group with healthcare professionals will conduct in presence in groups of up to 12 participants. It is planned to carry out two Focus Groups, one with professionals from the Azienda USL Toscana Nord Ovest and one with professionals from the IRCCS Stella Maris. The obtained results will allow the definition of innovative organizational models, including tele-assistance and tele-rehabilitation pathways, to be validated and thus integrated into the planning of the Regional Health Service (SSR).

### 2.2. Ethical Approval

The present study was approved by the Paediatric Ethics Section of the Tuscany Regional Ethics Committee (Italy), with study opinion registration number 303/2021, 30 November 2021. Written consent will be obtained from participants and/or their caregivers of eligible subjects after being verbally informed about the trial by the Principal Investigator or the research collaborators mentioned in the ethical committee-approved protocol. A child-friendly format will be provided for participants aged 7–14, too. Any pertinent modifications to the protocol will be promptly communicated to the aforementioned Ethical Committee for review and approval.

An appropriate electronic access system, secured with passwords, will be used for data collection and management. Conforming to the current privacy standards, each patient will be assigned a unique alphanumeric code. The record linking each child’s name to the assigned code will be securely maintained by the Principal Investigator.

In addition, the study has been registered in the Clinical Trial Protocol Registry and Results System (ClinicalTrials.gov, accessed on 22 January 2024, code: NCT06219447).

### 2.3. Sample Size

Based on the annual count of children referred to the recruiting centers for clinical management and rehabilitation, a final sample of 100 subjects is deemed sufficient for conducting a *t*-test to analyze differences in primary endpoints between groups, assuming an effect size (d) of at least 0.4, a significance level (α) of 0.05, and a power (1-β) of 0.8.

### 2.4. Participants

Participants will be selected from the databases of the Department of Developmental Neuroscience of the IRCCS Stella Maris (Pisa, Italy) and of the Azienda USL Toscana Nord Ovest (Italy). Eligible subjects and their families will subsequently receive invitations to participate in the study, and informed consent will be obtained from either the subjects and/or their caregivers before the enrolment. Subjects will be recruited according to the following specific inclusion criteria:

1. Congenital or acquired developmental disabilities;
2. Age between 6 and 30 years at the time of recruitment;
3. Cognitive functioning that allows sufficient understanding of proposed activities and cooperation in exercises investigated by appropriate rating scales (Wechsler Preschool and Primary Scale of Intelligence (WPPSI)-III, WPPSI-IV [37], Wechsler Intelligence Scale for Children (WISC)-IV [38], Wechsler Adult Intelligence Scale (WAIS)-IV [39], Leiter International Performance Scale—Revised Edition [40], Leiter International Performance Scale—Third Edition [41], Color progressive [42] or Standard progressive Raven matrices [43]);
4. Distance from the clinical center that allows periodic in-person evaluation of progress;
5. Internet access at home;
6. Caregivers able to be committed to and cooperate in an intensive rehabilitation home-based program;
7. Manual Ability Classification System (MACS) [44] level < V;
8. Subjects will be excluded in case of severe comorbidities (such as behavioral impairments, auditory and visual impairments) and/or severe cognitive disability that may preclude adequate cooperation in clinical assessment and during the tele-rehabilitation sessions.

As mentioned above, after the enrolment, participants will be stratified according to several criteria, including (i) diagnosis (congenital or acquired developmental disorder group), (ii) GMFCS level (GMFCS I-II/GMFCS III–IV), (iii) age (6–10/11–15/16–20/21–30), (iv) and technology utilized (APP vs. Home Kit). Randomization, sequence generation and preparation of group allocation materials will be carried out by an independent researcher who will not be involved in the trial. Using a computer-generated set of random numbers, they will be then allocated to experimental or control groups. Participants, parents, and clinical staff will be aware of the allocation group. Pairs will be randomly divided into two groups with a 1:1 experimental/SC (wait-list) ratio. Participants assigned to the experimental group will begin treatment with technology immediately for a 3-month period, while those in the SC group will continue with their usual care.

### 2.5. Study Procedure and Intervention

Subjects meeting the eligibility criteria will be directed to the research team for enrolment. All the comprehensive details about the study procedures will be provided to participants and/or their caregivers during an interview before obtaining informed consent. Thus, demographic and clinical measures will be collected. Beyond detailed assessment and procedures planned for the study will be conducted at IRCCS Stella Maris Foundation and Azienda USL Nord-Ovest.

For the study purposes, two different technologies will be implemented for the pediatric population and used for cognitive, motor, and language tele-rehabilitation: the VRRS Home Kit Tablet and the MedicoAmico APP. Since VRRS is one of the several home-based technologies available and has shown promising results in previous studies involving pediatric patients for various rehabilitation purposes, including speech and motor therapy [33,34], we decided to use this technology in this study.

#### 2.5.1. VRRS Home Kit Tablet

VRRS features a wide range of innovative non-immersive virtual reality medical devices, incorporating a multi-domain technology developed by Khymeia (an Italian Small–Medium Enterprise, SME) and tested for rehabilitation both in clinical settings and tele-rehabilitation. Utilizing biofeedback and augmented feedback mechanisms, VRRS enhances user engagement and compliance [45,46]. Initially designed for the adult population, recent applications with children and adolescents have shown promising preliminary outcomes [32,34,47,48].

**Figure 5:**
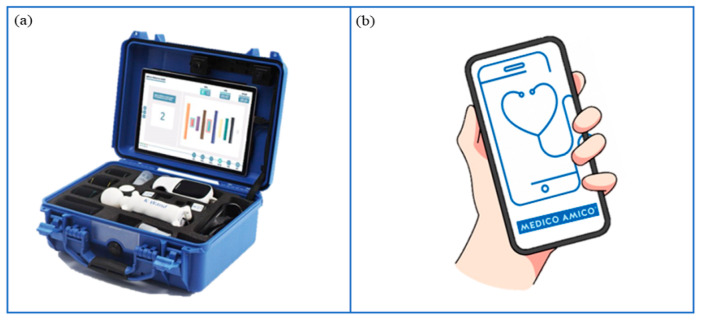
Technological devices for tele-rehabilitation purposes. (**a**) VRRS Home Kit Tablet: A case provided to patients and their families, equipped with a tablet and a set of integrated motion sensors, allowing to delivery of home tele-rehabilitation pathways. (**b**) MedicoAmico APP: A medical device directly downloadable to patients’ and families’ devices (computer, phone, tablet) to perform cognitive, speech-language, and motor interventions. Remotely monitored by the clinician.

Among different VRRS devices, VRRS Home Kit Tablet (Figure 5a) allows cognitive, language and motor rehabilitation to be delivered at home through tele-rehabilitation pathways. This system encompasses a tablet with a wide exercise library and different USB peripheral devices, such as balances and sensors (K-sensors, i.e., inertial measurement unit (IMU) sensors; K-wand) supporting motor rehabilitation across different body districts (i.e., upper and lower limbs, trunk). Moreover, touch screen activities enable neuropsychological and speech therapy sessions.

Considering that the VRRS Home Kit Tablet is currently mainly used for adulthood rehabilitation, during the project the library of exercises will be reviewed and re-organized, selecting those most suitable for the pediatric population, also considering the functional profile of different neurodevelopmental disabilities. Furthermore, activities will be enriched with options to customize the exercises’ difficulties, including self-progression of levels, duration, speed and amplitude of movement, with new scenarios to enhance engagement and interactivity for children.

#### 2.5.2. MedicoAmico APP

MedicoAmico APP (Figure 5b) is a medical device for tele-medicine and tele-rehabilitation, developed by Khymeia, downloadable on patients’ smartphones or tablets following General Data Protection Regulation (GDPR). The app comprises a library of cognitive, language and motor exercises that will be implemented during the project. Indeed, new packages of modular interactive exercises will be developed, featuring parameters including duration, repetitions, difficulty, speed, pace and amplitude of movement. The new exercises will include images and specific instructions tailored for children and adolescents, followed by a post-activity self-assessment rating.

**Figure 6:**
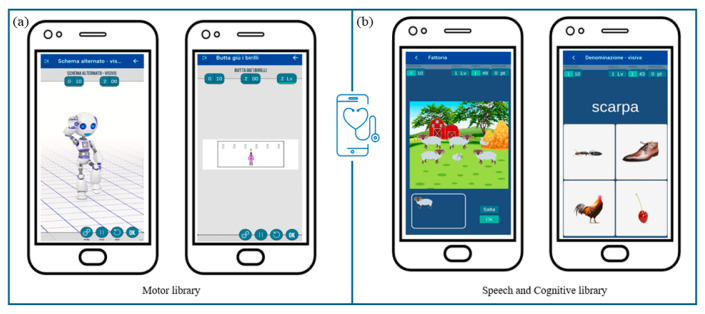
MedicoAmico APP exercises’ library. (**a**) Examples of motor activities implemented and now available on MedicoAmico APP. (**b**) Examples of cognitive and speech-communication exercises available within the wide library of the medical device. Parameters, including repetition, time and difficulty levels, are displayed in the upper section.

Specifically, within the motor library (Figure 6a), new exercises will be created to address the rehabilitation of global coordination, gross motor skills, and functional balance. These activities will include imitating movements performed by a robot-like avatar and searching materials to build motor paths guided by auditory and/or visual cues.

Concerning the speech and cognitive library (Figure 6b), current exercises will be reviewed and categorized based on neuropsychological processes (i.e., attention, visuospatial and verbal working memory, inhibition) and language skills (i.e., lexical and semantic skills, reading and writing skills) involved. Furthermore, the exercises will be modified to be more suitable for pediatric use, arranging additional settings and enabling a progression of increasing difficulty in line with self-advancement criteria. Within these libraries, activities most suitable for each subject will be identified, facilitating the development of customized treatment protocols.

#### 2.5.3. Treatment Procedure

The designated devices for tele-rehabilitation (VRRS Home Kit or MedicoAmico APP) will be delivered to participants’ homes. Moreover, a dedicated printed manual with instructions will be provided, also including all the contacts of both technical and clinical staff for remote assistance in case any problems occur during the treatment. This will follow a familiarization phase with clinicians explaining the system’s operating procedures to participants and their caregivers, including safety aspects.

The treatment will last for 3 months, with a frequency of about 2–3 sessions per week, according to the rehabilitation goals and users’ needs. Clinicians will consistently monitor the progression of the treatment using a dedicated web platform. The sessions would be performed in two modalities: (i) online, with clinicians interacting in real-time with the patient, also having the remote control of the system; (ii) offline, with the patient executing alone the training session before scheduled by clinicians.

During the initial period, clinicians will supervise the treatment online, guiding both patients and their families in system utilization. As patients and caregivers become more confident in using the system and according to treatment goals, online sessions will be decreased in favor of offline ones.

### 2.6. Outcome Measures

#### 2.6.1. Primary Outcome Measures

The acceptability and usability of ICT technologies in pediatric populations will also be assessed through the following Key Performance Indicators (KPIs), summarized in Table S1.

Service and technology KPIs:

- Study adherence: rate of acceptance of the participation in the study calculated as the number of eligible participants that agree to join the project;
- Adherence to training: total number of training sessions completed on the total number of sessions scheduled by the clinicians;
- Number of dropouts: number of participants that will not complete all of the study procedures;
- Number of sessions completed in the target time: total number of training sessions completed in the timeframe set by clinicians;
- Hardware and software technical problems: number of issues and malfunctioning experiments during the training sessions;
- Ad hoc feasibility questionnaires: according to the standard definitions of usability [49,50,51] and acceptability [52], specific items ranked on a five-point Likert scale (1 most negative, 5 most positive) will be defined. The aim will be to investigate four feasibility domains: (i) exercise customization, (ii) adaptability of home training, (iii) required effort and (iv) systems suitability. An introductory section will include both open and multiple-choice questions to collect information about the patient, the experimenter who fills out the questionnaire, and the devices and peripherals used. Three questionnaire versions will be available for different stakeholders (participants, parents, clinicians). The children’s format will be structured to be easy to understand for even the youngest participant and facial expressions shown through emoticons will help the children to assign the Likert-scale rank. Questionnaires will, therefore, be filled out at the end of the training by clinicians who administered the session, parents and participants over 6 years old and with cognitive and neuropsychological functioning, allowing full question comprehension and adequate cooperation;
- Medical Equipment Utilization: for each involved technology: the average time of utilization per session; the related consumables (if any)—the related power consumption;
- Set-up time: the time necessary for setting up the involved technologies;
- Training time/learning curve (for patients).

Operational KPIs related to the impact of tele-rehab on facilities’ performances (inpatient–outpatient and at-home rehab services):

- Average length of hospital-facility stays: self-explanatory;
- Patient wait time for the first rehabilitation session;
- Bed turnover: number of discharges in the given time period/number of beds in the facilities during that time period;
- Bed occupancy rate: number of occupied beds on the total beds;
- Readmission rate: total readmissions on the total number of patients;
- Number of patients treated: self-explanatory;
- Number of beds dedicated: self-explanatory;
- Staff to patient ratio: number of staff needed per patient per session;
- Space requirements (in square meters) of the facilities’ surface requested for the specific treatments;
- Training time/learning curve (for healthcare professionals or families) in supporting patients during the rehab sessions;
- Physiotherapists’ mental workload: the amount and complexity of information decisions physiotherapists have to manage or make per session.

#### 2.6.2. Secondary Outcome Measures

In order to obtain efficacy data, given the heterogeneity of the clinical population in terms of both chronological age and functional profile, a wide set of tests with normative data covering an extended age range have been defined. This approach will allow the selection of the tests most suited to each patient’s functional profile for the pre–post-training assessment of different cognitive, neuropsychological, motor, and language abilities, as detailed in Table S2. Moreover, the training’s impact on daily life functioning will be considered.

Specifically, the following outcome measures, grouped considering the function assessed, will be deemed.

- Attention: assessment of visual sustained and selective attention through different barrage tasks of increasing complexity (i.e., Leiter-3—Sustained attention subtest [41], BIA—CP subtest [53]);
- Visuomotor processing: assessment of the ability to integrate and coordinate visuo-perceptual and motor functions by coping geometrical figures or following a narrow path under time pressure (i.e., NEPSY II—Visuomotor precision subtest [54], VMI [55], TPV test [56]);
- Visuospatial processing: assessment of the ability to be oriented in visuospatial coordinates by judging lines’ orientation or locating a target on a map (i.e., NEPSY II—Arrow subtest, NEPSY II—Route finding subtest [54]);
- Working memory: assessment of the ability to recall and/or actively manipulate visuospatial and verbal information through the repetition of a sequence of targets in the same or reverse order (i.e., BVN—Digit Span [57,58]; BVS Corsi—Corsi block tapping test [59]);
- Daily life functioning: assessment of clinical behavior and executive functioning in daily life contexts and activities (i.e., CPRS [60], BRIEF—P and 2 versions [61,62]);
- Learning skills: assessment of reading and writing abilities, considering different tasks of increasing length and complexity (i.e., DDE 2—reading and writing subtests [63], MT-3 [64], Martini [65], BVSCO 2—text writing subtests [66]);
- Receptive language: assessment of grammar and vocabulary comprehension, using multiple-choice figurative tasks (i.e., PPVT-R [67], TCGB [68]);
- Walking: assessment of aerobic capacity and endurance by walking task (i.e., 6MWT [69]);
- Gross-motor function: assessment of gross-motor functions and praxis skills, considering performances in different areas (i.e., GMFM- 88 [70], MABC-2 [71], APCM-2 [72]);
- Static and dynamic balance: assessment of functional balance skills through a set of different tasks (i.e., PBS [73], FRT [74], TUG [75]);
- Upper limb: assessment of upper limb motor abilities through unimanual and bimanual tasks, but also considering daily life activities (i.e., BoHA [76], AHA [77], MA2 [78], BBT [79], ABILHAND-Kids [80]);
- Movement disorders: assessment of movement disorder and their impact on motor functions and daily life activities, considering different body regions at rest and while performing specific tasks (i.e., MD-CRS 4-18 R [81]).

Furthermore, treatment effectiveness will be examined using the Goal Attainment Scale (GAS) [82], a rehabilitation outcome measure, which is scored based on individual goals hypothesized and achieved throughout the intervention. Finally, the merging of these datasets together with primary outcome measures will contribute to the formulation of innovative organizational models, incorporating tele-assistance and tele-rehabilitation pathways.

The primary and secondary outcome measures will be acquired and collected through electronic Case Report Forms that collect data coming from both the online questionnaires on acceptability and usability and data on the performances (see the secondary endpoints) of the involved children collected and filled-in the eCRF by the healthcare professionals.

### 2.7. Statistical Analyses

Descriptive statistics (means, standard deviation, frequencies) will be investigated, analyzing potential baseline differences between groups explored and computing the p values. The normal distribution of the sample will be investigated through the Shapiro–Wilk test. Subsequently, a comprehensive analysis, both qualitatively and quantitatively, will be conducted on the questionnaire data gathered from various stakeholders, aiming to assess the usability and acceptability of the devices used. Statistical analysis, such as ANOVA or regression, will be carried out to determine differences based on age, diagnosis, functional profile (cognitive and motor outcomes), and devices used during tele-rehabilitation. Furthermore, inferential analyses will be carried out to assess pre–post-treatment differences, gain efficacy data, and also consider the different devices used during tele-rehabilitation pathways. Finally, flowchart diagrams will be created to illustrate the different organizational models identified. Statistical significance will be considered at p<.05. Statistical Package for Social Sciences (SPSS) (IBM Statistic) will be adopted for carrying out the statistical analyses.

## 3. Discussion

This paper provides a comprehensive overview of the background and the study protocol for the development of innovative organizational models for tele-rehabilitation within the region of Tuscany (Italy).

An evolution of tele-medicine and tele-rehabilitation in the Italian healthcare landscape has occurred, particularly after the COVID-19 pandemic [83], with the establishment of a National Tele-rehabilitation network in 2015 by the Italian Ministry of Health. This network, involving the main Research Institutes (i.e., IRCCS), aims to promote the sharing of clinical-scientific activities and data and the development of collaborative projects to improve the prevention, diagnosis and rehabilitation of specific diseases (neurological, neuro-psychiatric, and related disorders), including neurodevelopmental disorders [84].

However, tele-rehabilitation is mainly applied within research projects and centers of excellence, with limited practical implementation in local health services where continuity of care is crucial.

So far, the available literature emphasizes the potential advantages of tele-rehabilitation, including enhanced access for individuals residing remotely or facing physical limitations [85]. Benefits also encompass time and cost savings [13], improved treatment adherence [86] and frequency [14], and the maintenance of elevated satisfaction [16]. The use of innovative technologies, such as virtual reality, allows the possibility to insert rehabilitation programs within playful and interactive scenarios, further supporting treatment adherence and motivation, especially among children. Additionally, with no doubt, tele-rehabilitation facilitates continuous treatment monitoring through the collection of quantitative data [87].

The use of these technologies is widespread in Italy and other foreign countries [88,89,90] with promising results but also showing several limitations, such as small sample sizes, which hinder the relevance of findings for economic analysis, and resistance to change among healthcare professionals due to insufficient knowledge, limited experience, and low computer literacy among health care personnel. Moreover, there is a pressing need to establish standards for procedures, protocols and objectives related to tele-rehabilitation [12,25,91]. This concern is especially critical in the developmental age, where research is comparatively limited compared to the adult population.

The lesson learned from the COVID-19 pandemic is that we can only partially apply tele-rehabilitation to daily neuro-rehabilitation routines in increasing and improving the patient’s care. There is a widespread agreement on the fact that face-to-face therapeutic interactions are an absolutely irreplaceable element in the relationship between patients and healthcare providers; however, tele-rehabilitation could act as the missing pieces of the puzzle leading to an optimal continuity of care and support a greater treatment intensity [92]. Furthermore, recent study results report that clinicians and the general population exhibit apprehension about tele-rehabilitation [93].

Considering neurodevelopmental disabilities as involving the impairment of multiple cognitive, motor and speech functions, many technological systems used in tele-rehabilitation could enable the implementation of integrated and multi-domain treatments within a unified rehabilitative setting. Thanks to the hardware and software variability of technological devices, clinicians could, therefore, choose the most effective rehabilitation solutions within the different possibilities by tailoring treatments based on specific treatment goals. However, results presented by a recent systematic review [25] showed that studies predominantly focused on the tele-rehabilitation of a single domain, specifically neuropsychological and motor functions, with less emphasis on speech therapy; only a limited number of studies addressed the simultaneous rehabilitation of multiple abilities.

In this framework, our research specifically focuses on integrating tele-rehabilitation into local health services, which is crucial for ensuring continuity of care. Additionally, while most studies have primarily addressed single-domain rehabilitation, our approach aims to integrate multi-domain treatments, thus providing a more comprehensive care model.

By incorporating tele-rehabilitation into local health services, we aim to enhance the continuity and intensity of care, particularly benefiting patients with neurodevelopmental disorders, but also the quality of care and the health care system. Health policies could use these results to develop facilities that support wider implementation of tele-rehabilitation, ensuring integration with face-to-face interactions so as to optimize the use of resources and improve patient outcomes.

However, this project faces certain limitations, including the generalizability of results across different populations or settings. Future research should address these limitations by including larger and more different sample sizes and exploring the applicability of our findings in various healthcare environments.

To the best of our knowledge, the TOSCANA TABLET project represents the first project focused on implementing tele-rehabilitation programs directly at the local level, with a specific emphasis on engaging the pediatric population. The potential effects will be not only on the patient’s care concerning the care contents but also on creating new innovative approaches and fruitful networks among professionals as a new model of care, laying the foundation for future and innovative proposals. Consequently, our effort aims to pave the way for more robust and effective tele-rehabilitation solutions that enhance patient care and support the evolving landscape of healthcare.

## 4. Conclusions

In conclusion, this study protocol aims to develop innovative organizational models to promote continuity of care through tele-rehabilitation for neuro-psychomotor and language functions in individuals with congenital and acquired developmental disabilities, with a specific emphasis on pediatric patients with neurodevelopmental disorders within the region of Tuscany, Italy.

By focusing on organizational aspects, acceptability, usability, and the performance of ICT solutions, this study seeks to fill gaps in the current literature and enhance the practical application of tele-rehabilitation within local health services. This approach aims to improve the continuity and intensity of care, thereby improving the overall quality of the healthcare system. Furthermore, a multi-domain approach can provide a more comprehensive care model, offering significant benefits for patients with neurodevelopmental disorders.

Potential effects include not only enhanced patient care but also the development of innovative approaches and collaborative networks among healthcare professionals. This will establish a novel model of care, paving the way for future innovative proposals and supporting the evolving landscape of healthcare.

## Data Availability

The information provided in this study can be obtained by contacting the corresponding author. As of now, the data cannot be accessed publicly due to the enrolment and signing of the participants’ consent being in progress. Furthermore, our research primarily focuses on methodology, and we do not possess any clinical data regarding the patients involved.

## Supplementary Materials

The following supporting information can be downloaded at: https://www.mdpi.com/article/10.3390/jcm13144159/s1, Table S1: Primary outcome measures summarizing Key Performance Indicators (KPIs); Table S2: Clinical measures for pre–post assessments of cognitive, neuropsychological, motor, and language skills.

## List of Abbreviations

Information and Communication Technologies (ICT); Italian Society of Child and Adolescence Neuropsychiatry (SINPIA); Italian Society of Physical and Rehabilitation Medicine (SIMFER); Italian Society of Neurological Rehabilitation (SIRN); Virtual Reality Rehabilitation System (VRRS); Gross motor function classification system (GMFCS); Experimental group (EG); Waitlist group (WG); Regional Health Service (SSR); Wechsler Preschool and Primary Scale of Intelligence (WPPSI); Wechsler Intelligence Scale for Children (WISC); Wechsler Adult Intelligence Scale (WAIS); Manual Ability Classification System (MACS); Small Medium Enterprise (SME); Inertial measurement unit (IMU); General Data Protection Regulation (GDPR); Key Performance Indicators (KPIs); Italian Battery for ADHD (BIA); Test Of Visuo-Motor Integration (test of VMI); Behaviour Rating Inventory of Executive Function (BRIEF); Battery for Neuropsychological Evaluation in Neurodevelopmental age (BVN); Battery for assessment of Visuo-Spatial Working Memory (BVS); Conners’ Rating Scales (CPRS); Dyslexia and Developmental Dysorthography Evaluation-2 (DDE-2); Battery for the Assessment of Writing and Orthographic Competence-2 (BVSCO-2); Peabody Picture Vocabulary Test—Revised (PPVT-R); Children’s Grammatical Comprehension Test (TCGB); Gross Motor Function Measure-88 (GMFM-88); 6 minutes’ walk test (6MWT); Functional Reach Test (FRT); Paediatric Balance Scale (PBS); Timed up and go (TUG); Assisting Hand Assessment (AHA); Both Hand Assessment (BoHA); Melbourne Assessment 2 (MA2); Box and Block (BBT); Movement Disorders—Childhood Rating Scale 4-18 revised (MD-CRS 4-18 R); Test of Visual Perception and Visuo-motor Integration (TPV); Movement Assessment Battery for Children- Second Edition (MABC-2); Praxic and Motor Coordination Skills-2nd Edition (APCM-2); Goal Attainment Scale (GAS); Statistical Package for Social Sciences (SPSS); electronic Case Report Forms (eCRF).
