# Supplementary material for: TABLET TOSCANA to Develop Innovative Organizational Models for Tele-Rehabilitation in Subjects with Congenital and Acquired Developmental Disabilities: A Wait-List Control Group Trial Protocol
Source: J Clin Med. 2024 Jul 16;13(14):4159. doi: 10.3390/jcm13144159 (PMC11277580; doi:10.3390/jcm13144159)
Supplement: Supplementary file 1 [file jcm-13-04159-s001.zip › jcm-3053744-supplementary.pdf]

Journal of
Clinical Medicine

MDPI

Table S1. Primary outcome measures summarizing Key Performance Indicators (KPIs).

Primary outcome measures	
**Service and technology KPIs**	**Operational KPIs**
Study adherence	Average length of hospital-facility stay
Adherence to training	Patient wait time for the first rehabilitation session
Number of dropouts	Bed turnover
Number of sessions completed in the target time	Bed occupancy rate
Hardware and software technical problems	Readmission rate
Ad hoc feasibility questionnaires – participants	Number of patients treated
Ad hoc feasibility questionnaires – parents	Number of beds dedicated
Ad hoc feasibility questionnaires - clinicians	Staff-to-patient ratio
Medical Equipment Utilization	Space requirements for the specific treatments
Set-up time	Training time/learning curve
Training time/learning curve	Physiotherapists mental workload

Table S2. Clinical measures for pre-post assessments of cognitive, neuropsychological, motor, and language skills.

Assessed function	Test	Detailed description	Age range
Attention	Leiter 3, Sustained Attention subtest	Assessment of visual sustained attention through symbols barrage tasks of increasing complexity within a set time frame.	3–75+
	Italian Battery for ADHD (BIA), CP subtest	Assessment of visual sustained attention through sequence of letters barrage tasks of increasing complexity.	7–13
Visuomotor and perceptual processing	NEPSY-II, Visuomotor precision subtest	Assessment of the ability to follow a narrow path with the pencil accurately under time pressure.	3–12
	VMI, Visuomotor Integration subtest	Assessment of the ability to integrate and coordinate visual perceptual and motor (finger and hand movement) functions, requiring copying geometrical figures.	3–18
	TPV, Visual perceptual and visuomotor integration test	Assessment of visuoperceptual and visuomotor integration skills through copying/reproduction, visual/motor speed, hand-eye coordination and spatial relationship tasks.	4–10
Visuospatial processing	NEPSY-II, Arrows subtest	Assessment of the ability to judge lines' orientation, considering and estimating direction, in distance and angularity of arrows arranged around a target.	5–16

	NEPSY-II, Route finding subtest	Assessment of the ability to use a small schematic map to locate a target on a more complex one, therefore orienting in visuospatial coordinates.	5–12
Working memory	BVN, Digit Span subtest	Assessment of short-term and working memory abilities in the verbal domain through the repetition of a sequence of numbers of increasing length, following the same or reverse order.	5–18
	BVS-Corsi, Corsi Block Tapping Task subtest	Assessment of short-term and working memory abilities in the visuospatial domain through the repetition of a sequence (of increasing length) of blocks tapped by examiners, following the same or reverse order.	6–13
Daily life functioning	CPRS	Assessment of clinical behaviors in children, with a specific focus on ADHD symptoms through a parent report questionnaire.	3–17
	BRIEF-P/2	Assessment of executive functioning in daily life contexts through a parent report questionnaire.	2–18
Learning skills	DDE-2, Word and non-word reading subtest	Assessment of reading abilities of a list of word and non-word items.	7–14
	DDE-2, Word and non-word writing subtest	Assessment of writing abilities of a list of word and non-word items under dictation.	7–14
	Martini, reading and writing tasks	Assessment of reading and writing skills considering flat bisyllabic and trisyllabic words, bisyllabic and trisyllabic words with consonantal groups.	6–8
	BVSCO-2, text writing subtests	Assessment of writing abilities through text writing under dictation and spontaneous text writing based on figure stories.	6–14
	MT3, reading and comprehension	Assessment of reading and comprehension skills of narrative and/or descriptive texts.	6–14
Receptive language	PPVT-R, Receptive vocabulary test	Assessment of lexical comprehension using nouns, verbs, and adjectives of increasing difficulty.	3–11
	TCGB, Grammatical comprehension test	Assessment of grammatical comprehension in a multiple-choice figurative test.	3–8
Walking	6MWT	Assessment of aerobic capacity and endurance considering the distance	2–65+

		covered over a time of 6 minutes by walking.	
Gross motor function	GMFM-88	Assessment of gross motor function according to five dimensions: Lying and Rolling, Sitting, Crawling and Kneeling, Standing and Walking, Running and Jumping.	0–16
	MABC-2	Assessment of movement difficulties in manual dexterity, aiming and catching and balance tasks.	3–16
	APCM-2	Assessment of motor and praxis skills and early detection of motor-praxis coordination deficit.	2–8
Static and dynamic balance	PBS	Assessment of functional balance skills in children with mild to moderate motor impairments.	5–15
	TUG	Assessment of mobility, balance, walking ability, and fall risk measuring the time it takes for the patient to stand up from a chair, walk 3 meters, turn around, walk back, and sit down.	3–18
	FRT	Assessment of dynamic balance evaluated in one simple task in a standing position measuring the maximum distance an individual can reach with an anterior shift with a fixed base of support.	5–75+
Upper Limb	AHA	Assessment of the assisting hand performance in children with unilateral upper limb disabilities during bimanual playful tasks.	1–18
	BoHA	Assessment of both hands abilities in children with bilateral cerebral palsy during bimanual playful tasks.	1–12
	ABILHAND-Kids	Assessment of manual abilities of children with cerebral palsy with a particular reference to independence in achieving some goals of everyday life.	6–15
	MA2	Assessment of unilateral upper extremity quality of movement in children with neurological impairments according to four elements: range of movement, accuracy of r each and placement, dexterity of grasp, release and manipulation and fluency of movement.	2–15
	BBT	Assessment of unilateral gross manual dexterity considering the number of	3–65+

		blocks carried from one compartment to the other in one minute.	
Movement disorders	MD-CRS 4-18 R	Assessment of movement disorders and their influence on daily life activities and on motor function in different regions of the body at rest and while performing specific tasks.	4–18
